# Prevalence of Pediatric Onset Multiple Sclerosis in Saudi Arabia

**DOI:** 10.1155/2021/4226141

**Published:** 2021-11-09

**Authors:** Reem Bunyan, Ghada Al Towaijri, Hessa Al Otaibi, Abid Kareem, Hussein Algahtani, Mousa Al Mejally, Ali Almubarak, Saad Alrajeh, Edward Cupler, Shireen Qureshi, Sadaga. Alawi, Mamdouh Kalakatawi, Yaser Al Malik, Shahpar Nahrir, Adel Alhazzani, Ashraf El-Metwally, Sahar Shami, Samah Ishak, Hajer Almudaiheem, Ahmed Al-Jedai, Mohammed AlJumah

**Affiliations:** ^1^Neurosciences Center, King Fahd Specialist Hospital (KFSH)-Dammam, Dammam, Saudi Arabia; ^2^Neurology Department, King Fahd Medical City Hospital (KFMC), Riyadh, Saudi Arabia; ^3^Neurology Department, King Fahd General Hospital-Jeddah, Jeddah, Saudi Arabia; ^4^Neurology Department, King Fahd General Hospital-Al-Madinah, Madinah, Saudi Arabia; ^5^Neurology Department, King Abdul-Aziz Medical City, Jeddah, Saudi Arabia; ^6^Neurology Department, Hera General Hospital, Makkah, Saudi Arabia; ^7^Neurology Department, Qatif Central Hospital, Qatif, Saudi Arabia; ^8^Neurology Department, Dr. Sulaiman Al Habib Hospital-Olaya Branch, Riyadh, Saudi Arabia; ^9^Department of Neurosciences, King Faisal Specialist Hospital & Research Center, Jeddah, Saudi Arabia; ^10^Neurology Department, Johns Hopkins Aramco Healthcare Company (JHAH), Dhahran, Saudi Arabia; ^11^Neurology Department, Prince Sultan Military Medical City, Riyadh, Saudi Arabia; ^12^Neurology Department, Al Nour Specialized Hospital, Makkah, Saudi Arabia; ^13^College of Medicine, King Saud bin Abdulaziz University for Health Sciences, Riyadh, Saudi Arabia; ^14^Neurology Division, King Abdul-Aziz Medical City, Riyadh, Saudi Arabia; ^15^King Abdullah International Medical Research Center, Riyadh, Saudi Arabia; ^16^King Saud Medical City, Riyadh, Saudi Arabia; ^17^College of Medicine, King Saud University, Riyadh, Saudi Arabia; ^18^College of Public Health and Health Informatics, King Saud Bin Abdulaziz University for Health Sciences, Riyadh, Saudi Arabia; ^19^Itkan Health Consulting, Riyadh, Saudi Arabia; ^20^Deputyship of Therapeutic Affairs, Ministry of Health, Riyadh, Saudi Arabia

## Abstract

**Background:**

The prevalence of multiple sclerosis (MS) appears to be increasing worldwide. However, data on the pediatric onset of MS is lacking, particularly in developing countries.

**Objective:**

This study is aimed at reporting the current burden of the pediatric onset of MS in the five regions of Saudi Arabia.

**Methods:**

This study used relevant data from the National Saudi MS Registry that was operational between 2015 and 2018. The data on patients with pediatric onset MS from all the hospitals included in the registry was retrospectively analyzed using the age of diagnosis. Patients who were 1-18 years old when diagnosed were included in the analysis.

**Results:**

The registry included 287 patients with pediatric onset MS, with a mean age of diagnosis at 15.7 (SD: 2.06). 74.2% of the participants were females. For the included hospitals, the estimated prevalence of pediatric MS was at 2.73/100,000 pediatric Saudi population. The prevalence of pediatric MS in the remaining nonparticipant hospitals was then projected taking into account both the size of pediatric population in the Kingdom per region and the number of facilities treating and managing MS in each of the corresponding regions. The overall projected prevalence was found to be 14.33/100,000 Saudi pediatric population.

**Conclusion:**

To the best of our knowledge, this study reported the latest epidemiological data of pediatric onset of MS in Saudi Arabia. The current prevalence of MS among the pediatric Saudi population was found to be 2.73/100,000, and the overall projected prevalence was estimated at 14.33/100,000. Our findings were similar to those in other pediatric MS cohorts. Further studies are needed to understand the long-term prognosis, response to treatment, and disease course.

## 1. Introduction

Multiple sclerosis (MS) is recognized as a disease affecting predominantly adults. However, in the 1980s, pediatric onset of MS became recognized, and more attention has been directed towards the disease in this age group, its diagnosis, and management [1]. A recent review of published data on pediatric onset of MS during 1965-2018 reported that incidence of pediatric onset of MS is 0.05-2.85/100,000, and the prevalence is 0.69-26.92/100,000 [2].

The age of onset of MS typically ranges between 20 to 40 years; however, pediatric onset of MS is known to occur before the age of 18 years [2–5]. Pediatric onset multiple sclerosis occurs during the “key formative years of education and during the period of active brain maturation” [6]. Findings from number of studies suggest that it may have lasting consequences on affected individuals and potentially disrupting their ability to effectively study, work, and engage in society, emphasizing the need for understanding and discovery of specific clinical or demographic risk factors and eventually protective and supportive strategies for all patients with multiple sclerosis [6, 7].

Various factors are associated with the pediatric onset of MS. Exposure to viruses, such as Epstein-Barr virus (EBV), has been reported in children that later developed MS. Other factors include childhood obesity, vitamin D deficiency, and metabolic syndrome [8, 9]. Genetic factors have also been implicated in pediatric onset MS as in adult onset MS [10, 11].

The predominant clinical features of pediatric onset MS are headache, nausea, vomiting, fever, seizures, an altered level of consciousness, motor, sensory, and cerebellar and brainstem dysfunction [1]. Studies have reported good recovery among children compared to adults, with the majority recovering after their first attack and very few children suffering from disability [12, 13].

In Saudi Arabia, adult onset MS is more systematically studied; however, there is a shortage in the epidemiology of pediatric onset of MS. This article is aimed at reporting the prevalence of pediatric onset MS using data from the National Saudi MS Registry, in addition to describe relevant disease characteristics in that population, such as symptoms at onset, disease course, and family history which in turn may provide valuable information to better understand the actual burden of the disease, the risks for development, and progression. Using national hospital-based registries and databases to report burden of disease is known to help future planning and policymaking at both levels, regional and national [14]. Despite the low prevalence of pediatric onset MS globally, it is important to understand its epidemiology in the Kingdom as it could help in decision-making and various clinical practices.

## 2. Methods

The study utilized data collected earlier by the National MS Registry that was launched in 2015 and lasted until 2018. Detailed description of the registry can be found elsewhere [14, 15]. Physicians in participating hospitals entered patients' disease related data into a web-based application. Data on people diagnosed with MS at the age of 18 year or younger (≤18 years) were included in this study.

The frequency distribution tables were generated according to the five major regions in the Kingdom (Western, Central, Eastern, Southern, and Northern Regions). Demographics, clinical characteristics, affected CNS regions, and family history were summarized using descriptive statistics, such as mean (SD), median (interquartile range), or frequency (percentage).

Projections for the overall prevalence were performed at regional and national levels, following the same methodology used in our previously published paper that described the overall prevalence of the disease in KSA and utilizing the same registry database [14]. The overall projected prevalence of pediatric onset MS in the Kingdom was calculated and extrapolated taking into account the number of registered patients with pediatric onset MS in the included hospitals in the registry (20 hospitals) in each region and assuming a similar number of cases in the remaining nonparticipant hospitals for the same region, in addition to the total number of pediatric population in each region [14].

The total pediatric population in Saudi Arabia (the denominator for projected overall prevalence) was last recorded at 10533635 in 2018 [16]. Finally, it is noteworthy to point out that the regional actual and projected prevalence were calculated based on the patients' region of residence rather than the hospitals region where they were registered.

## 3. Results

The Saudi National Registry described in a previous study [14] included 287 patients with pediatric onset MS from the different regions of the Kingdom. The median age of MS diagnosis was 16.0 years (IQR: 15.0-17.0), and 74.2% of patients were females. For the included hospitals in the registry, the prevalence of pediatric MS was estimated at 2.73/100,000 pediatric Saudi population. Based on these estimates, the prevalence of pediatric MS in the remaining nonparticipant hospitals was then projected considering both the size of pediatric population in the Kingdom per region and the number of facilities treating and managing MS patients in each of the corresponding regions as shown in Table 1.

Finally, the overall prevalence was estimated and found to be 14.33/100,000 Saudi pediatric population as shown in Table 2. The prevalence of pediatric onset MS varied between the different regions in the Kingdom and was found to be the highest for the western region. Projected prevalence with respect to regions was found to be different, with the highest prevalence reported in the eastern region followed by the western, northern, central, and southern.

Out of the total number of patients with pediatric onset MS, 39.4% were from the western region, 34.1% from the central followed by 17.4% from the east, 5.2% from the south, and 3.8% from the northern region as shown below in Table 3.

Table 4 shows that the most common symptoms at onset as reported by participants were motor weakness (55.1%) followed by visual and sensory symptoms (50.5% and 41.8%, respectively). Among all patients, the majority had a polysymptomatic presentation with 69.3% presenting with two symptoms, 10.3% with three, 2.4% with four, and 1.2% with five. The CNS region affected was documented among 85.3% of patients at the time of enrolment in the registry, and the most commonly affected region was the cerebellar (31.0%) followed by the visual and spinal pathway (28.9% and 20.9%, respectively). Concomitant diseases were reported among 9.8% of patients.

**Figure 1:**
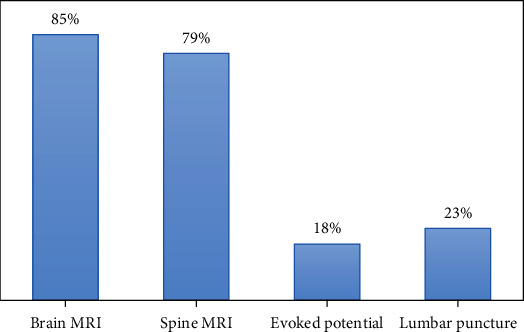
Diagnostic tests among individuals with pediatric onset of MS (*n* = 287).

Brain MRI scans were carried out for 85.4% of the patients, spine MRI in 79% (cervical MRI for 35.5%, whole spine MRI for 29.3%, and thoracic MRI for 14.3%) as seen in Figure 1 below. Evoked potentials were carried out in 17.8% of patients and lumbar puncture in 23.3%. Of the total, 38.0% patients had no history of relapse, 87.7% were using medication for relapse, and 80.0% were taking disease-modifying therapy (DMTs).

**Figure 2:**
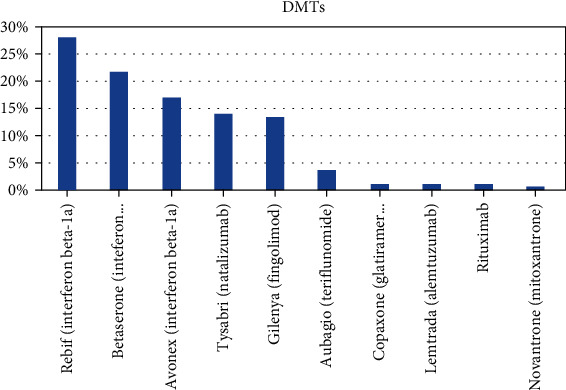
DMTs among patients with pediatric onset of MS (*n* = 232).

Among those on DMTs, majority used Rebif (interferon beta-1a) (28.0%), followed by Betaserone (interferon beta-1b) (21.6%), Avonex (interferon beta-1a) (16.8%), Tysabri (natalizumab) (13.8%), and Gilenya (fingolimod) (13.4%) as shown in Figure 2.

Of the total patients enrolled in the registry, 94.0% were diagnosed with relapsing remitting MS. The mean EDSS score reported at the time of inclusion was 1.67 (SD: 2.12).  Table 5 shows that family history of MS was present among 17.5% of patients, with parental consanguinity among 33.9%, and 7.4% reporting that their siblings were also affected with MS.

## 4. Discussion

To the best of our knowledge, this is the first study to provide the current estimates of prevalence of pediatric onset of MS in Saudi Arabia. An earlier study by AlJumah, Bunyan, Al Otaibi et al. (2020), which was derived from the same registry, reported the overall prevalence of MS in the Kingdom and projected the overall prevalence to be 40.40/100,000 population, while the current study presents preliminary findings specific to the burden of pediatric onset of MS in the Kingdom and projected the pediatric onset overall prevalence to be 14.33/100,000 Saudi pediatric population. Although it has been established that the prevalence of multiple sclerosis (MS) seems to be increasing worldwide, data on the pediatric onset of MS is still lacking in general.

MS is a disease that mainly affects adults, but it can also affect children. Globally, the incidence and prevalence of pediatric onset of MS are low; however, an increase in the rates of both the incidence and prevalence of the pediatric onset of MS has been reported [5]. Thus, this article is aimed at reporting the current epidemiology in Saudi Arabia as a whole and at a regional level of the country.

The current prevalence reported in our study estimated at 2.73 was much lower than that reported from the Kuwaiti registry, which was estimated at 6.0/100,000 [17], however, closer to prevalence reported in other studies [2]. The median age of onset of our cohort was 15.0 years, which was quite similar to what was seen in Kuwait (15.4 years), but higher than what was reported in the western world. For example, the UK reported that the mean age of onset was 9.7 years, while Canada reported 10.5 years and France 9.9 years [17–20]. Female predominance was reported globally and regionally, which was supported by the findings in this study, in which two thirds of the patients were females [17–20].

Variation in the findings could be related to the high rates of MS internationally, evolution of the diagnostic criteria, the use of surveillance data, and other methodological variations. The findings of this study were extracted from the data of a well-managed registry that included data from all regions of Saudi Arabia. Likewise, the suggested upward trend in the prevalence of the pediatric onset of MS in Saudi Arabia could have several explanations. Firstly, the overall increasing trend in the prevalence of pediatric onset MS globally, secondly, the increased awareness and improved diagnostic tools that may have helped in the early detection and identification of these patients, and lastly, the establishment of the national registry that has helped in the proper identification and reporting of cases, thus maintaining a record for reporting true estimations.

However, the reported prevalence in our study should be concluded cautiously for multiple reasons. For instance, the registry included data from 19% of hospitals treating the MS population in the Kingdom. In addition, the projected prevalence was estimated under the assumption that the prevalence will be the same across the remaining hospitals, which could impact the findings by slightly under or overestimating the true picture. Finally, the pediatric onset of MS was reported retrospectively by using the date of diagnosis, which in turn could affect the actual burden of the disease. Despite all these limitations, the study showed the current estimates and provided important insights that may help policy/decision makers to better address the potentially growing rates of pediatric MS in the Kingdom.

Overall, the rates of the pediatric onset of MS in Saudi Arabia are not significantly higher when compared with western countries where the prevalence of MS is generally high. However, an increasing trend could be seen throughout the country in the future. Gender disparity and other signs and symptoms should be kept in consideration while diagnosing and treating pediatric onset of MS. Through this study, we were able to provide initial epidemiological data and insights on pediatric onset MS in Saudi Arabia and the prevalence at a regional level and country level. These findings could be the baseline foundation for future studies and an array of future research initiatives. Further longitudinal studies targeting the long-term outcomes, relapse, and management are warranted to build upon the findings of this study.

Our study showed the median age at presentation to be 15.0 years similar to findings in other pediatric MS cohorts in the postpubertal age, with only 4.9% of patients younger than the age of 12 years. The Saudi Health Council defines the pediatric age group to be from birth up to 14 years and adolescence from 14 to 18 years. There are also variations in the age at which patients' transition from pediatric to adult care providers. These factors might influence patterns in diagnosis and care for pediatric onset MS in Saudi Arabia. Further studies are needed to assess this variation within the Kingdom's regions and among respective hospitals.

The clinical phenotype found in our study was similar to that in other pediatric MS cohorts with a common polysymptomatic presentation. The most common initial symptom of the disease was motor weakness in 55.1% of patients, followed by visual symptoms in 50.5%, and sensory symptoms in 41.8%. The most common CNS region affected initially was cerebellar, followed by visual in 28.9%. These findings are also similar to those in the literature from other pediatric cohorts. None of the patients reported having symptoms related to altered level of consciousness, seizures, or a prior diagnosis of acute disseminated encephalomyelitis (ADEM). The mean EDSS score at the time of enrollment in the registry was low. Longitudinal studies are needed to study disability among these patients and how they compare with adult onset disease.

## 5. Conclusion

This study reported the epidemiology of pediatric onset of MS in Saudi Arabia utilizing a national disease registry. The current prevalence of MS among the pediatric Saudi population was found to be 2.73/100,000, and the overall projected prevalence was estimated at 14.33/100,000. Our findings were similar to those in other pediatric MS cohorts. Further studies are needed to understand the long-term prognosis, response to treatment, and disease course.

### 5.1. Study Limitation

Data on MS patients was not collected from all hospitals diagnosing and treating MS population in the Kingdom; however, the included hospitals in the registry were comparable in their characteristics to those not included (such size, catchment area, number of beds, average number of neurologists). Therefore, the prevalence of pediatric MS was a projection based on the assumption that number of diagnosed MS cases in included/participating hospitals in the registry (in each region) is similar to those in nonparticipating hospitals in the same region. While this limitation may impact the generalizability of the findings, nevertheless, the study represents a good initial effort in establishing current estimates of prevalence of pediatric onset of MS in Saudi Arabia. Further and more comprehensive studies are warranted to get more generalizable results.

## Figures and Tables

**Table 1:** Total Saudi pediatric population, number of facilities treating MS patients, and number of facilities included in the National MS Registry, Saudi Arabia.

Regions	Pediatric population	Hospitals/facilities treating MS patients in KSA	Hospitals/facilities included in registry
Western region	3387191	30	7
Central region	3047059	36	7
Eastern region	1516795	22	4
Southern region	1694334	9	2
Northern region	888256	8	0
Total “2018”	10533635	105	20

^∗^Table adapted from AlJumah, M., Bunyan, and R., Al Otaibi, H. et al. (2020).

**Table 2:** Total Saudi pediatric population and prevalence of pediatric MS among Saudi pediatric population.

Regions	Total Saudi pediatric population	Patients with pediatric onset MS (from participant hospitals^∗^)	Prevalence/100,000 pediatric population	Projected prevalence/100,000 population
East	1516795	50	3.30	18.13
West	3387191	113	3.31	14.71
North^∗∗^	888256	11	1.24	9.91
Central region	3047059	98	3.22	16.56
South	1694334	15	0.89	3.98
Total “2018”	10533635	287	2.73	14.33

^∗^20 hospitals participated in the registry. ^∗∗^Given that there were no participating hospitals from the northern region in the registry, the assumption was made that the 11 patients enrolled in the registry from the northern region were referred to hospitals in other regions.

**Table 3:** Baseline characteristics of individuals with pediatric onset of MS (*n* = 287).

Demographics	Frequency	Percentage
Age		
Median IQR	16.0 (15.0-17.0)	
<12 years	14	4.9
≥12-18 years	273	95.1
Gender		
Male	74	25.8
Female	213	74.2
Regions		
West	113	39.4
Central	98	34.1
East	50	17.4
North	11	3.8
South	15	5.2

**Table 4:** Signs and symptoms and the involvement of CNS region among individuals with pediatric onset MS (*n* = 287).

Symptoms and affected regions	Frequency	Percentage
Symptoms of 1^st^ attack		
Motor weakness	158	55.1
Bulbar symptom	5	1.7
Bladder and bowel difficulty	18	6.3
Neuropsychological function	11	3.8
Ataxia	79	27.5
Sensory symptoms	120	41.8
Visual symptoms	145	50.5
Fatigue	38	13.2
Pain	25	8.7
Seizures	8	2.8
CNS region affected		
Any region affected	243	85.3
Visual pathway	83	28.9
Spinal cord	60	20.9
Brainstem	43	15.0
Cerebellar	89	31.0
Pyramidal tract	30	10.5
Bowel	17	5.9
Sensory function	44	15.3
Multiple regions	64	22.3

**Table 5:** Family history of MS among individuals with pediatric onset of MS (*n* = 287).

Family history	Frequency	Percentage
Family history of MS^∗^		
Yes	50	17.5
No	235	82.5
Parental consanguinity^∗^		
Yes	92	33.9
No	179	66.1
Sibling affected^∗^		
Yes	19	7.4
No	239	92.6

^∗^ indicates missing data.

## Data Availability

Due to the nature of the data involved in this study, access to this data is restricted and requires appropriate approvals from the authorized and participating parties.

## Abbreviations

MS: Multiple sclerosis
KSA: Kingdom of Saudi Arabia
EDSS: The Expanded Disability Status Scale
CNS: Central nervous system
ADEM: Acute disseminated encephalomyelitis.
